# FACTORS ASSOCIATED WITH HOME CARE AIDES' JOB SATISFACTION DURING COVID-19

**DOI:** 10.1093/geroni/igac059.630

**Published:** 2022-12-20

**Authors:** Richard Chunga, Verena Cimarolli, Natasha Bryant, Kathrin Boerner, Robyn Stone

**Affiliations:** University of Massachusetts Boston, Boston, Massachusetts, United States; LeadingAge, Washington, District of Columbia, United States; LeadingAge, Washington, District of Columbia, United States; University of Massachusetts Boston, Boston, Massachusetts, United States; LeadingAge, Washington, District of Columbia, United States

## Abstract

The COVID-19 pandemic has pronounced already high turnover rates among home care aides (HCAs). High turnover negatively affects home care quality. Job satisfaction among HCAs is a major driver of turnover, but little is known about factors associated with HCAs’ job satisfaction during COVID-19. Using survey data, the purpose of this study was to identify variables associated with job satisfaction among HCAs during the pandemic (N=114). Correlational analyses show that lower job satisfaction is associated with experiencing financial hardship, lack of PPE and employer protocols/guidance, understaffing, lower quality of employer communication related to COVID-19, and HCAs’ lower perceived preparedness to care for clients with COVID-19. In a regression analysis, experiencing financial hardship and lower quality of employer communication remained significant predictors. Findings underscore the importance of employer supports in HCAs’ job satisfaction and provide important lessons for how employers can support HCAs during the pandemic and beyond.

